# Supplementary material for: Patterns and Determinants of Essential and Toxic Elements in Chinese Women at Mid-Pregnancy, Late Pregnancy, and Lactation
Source: Nutrients. 2021 Feb 19;13(2):668. doi: 10.3390/nu13020668 (PMC7922563; doi:10.3390/nu13020668)
Supplement: Supplementary file 1 [file nutrients-13-00668-s001.zip › nutrients-1099395-supplementary.pdf]

Table S1. Data on the Certified Reference Materials (CRM) and limit of detection (LOD).

Elements	CRM[1]					LOD (ng/mL)
	Measured values [1]				Standard values	
	Value 1	Value 2	Value 3	Mean ± SD		
Cu[2]	1229	1204	1244	1226 ± 20	1220 (976 – 1460)	0.03
Fe[2]	1108	1132	1142	1128 ± 17	1110 (888 – 1330)	0.9
Zn[2]	1531	1586	1532	1550 ± 32	1540 (1230 – 1850)	0.06
Cr[2]	11.3	10.6	10.4	10.8 ± 0.5	10.6 (8.5 – 12.7)	0.002
I[2]	96.8	97.5	97.1	97.1 ± 0.4	98.4 (78.7 – 118.0)	0.03
Mn[2]	14.7	14.6	15.0	14.8 ± 0.2	15.5 (12.4 – 18.6)	0.008
Co[2]	9.29	9.37	9.38	9.35 ± 0.05	9.66 (7.73 – 11.60)	0.001
Mo[2]	6.97	6.96	6.44	6.79 ± 0.30	6.32 (5.06 – 7.58)	0.002
B[3]	0.6	0.6	0.7	0.6 ±0.06	0.6 (Reference value)	0.005
Ni[2]	13.3	13.3	13.8	13.5 ± 0.3	13.9 (11.1 – 16.7)	0.005
Al[2]	57.7	54.0	52.0	54.5 ± 2.9	55.6 (41.7 – 69.5)	0.1
Sb[2]	6.17	6.07	6.09	6.11 ± 0.05	5.93 (4.74 – 7.12)	0.003
Hg[2]	9.51	9.62	9.37	9.50 ± 0.13	9.44 (7.55 – 11.30)	0.007
Cd[2]	10.37	10.85	10.29	10.51 ± 0.30	10.70 (8.56 – 12.80)	0.003
Pb[3]	0.13	0.13	0.12	0.12 ± 0.00	0.12 (0.09 – 0.15)	0.012

[1] The measured values were obtained from three repeated measurements of CRM; [2] The elements used standard plasma samples (ClinChek®-Control Plasma Control，Level II: 8884) for quality control, with a unit of ng/mL; [3] The elements used pork liver samples (GBW10051) for quality control, with a unit of μg/g.

Table S2. Dietary intakes for participants (n = 1208) [1].

Food items	Mean (SD)				Classification of participants			
	Overall	Geographic area			Lowest and middle tertiles		Highest tertile	
		Coastland	Inland	Lackland	Mean (SD)	*n* (%)	Mean (SD)	*n* (%)
Freshwater aquatic product, g/d	43.4 (88.8)	36.0 (93.0)	25.7 (59.2)	68.6 (102.9)	10.9 (10.1)	811	109.6 (131.3)	397
Marine aquatic product, g/d	29.7 (46.0)	62.5 (62.0)	14.2 (24.6)	12.9 (18.6)	6.9 (6.8)	804	75.1 (56.0)	404
Mutton, g/d	9.3 (42.3)	2.8 (13.6)	24.9 (69.0)	0	0.03 (0.2)	813	28.5 (70.3)	395
Formula, tsp /d	0.7 (2.1)	0.3 (1.3)	0.2 (1.0)	1.7 (3.0)	0	966	3.7 (3.3)	242

[1] Dietary information was not collected for three participants, therefore 1208 were included in the analyses for dietary intakes.

Table S3. Dietary reference intakes of essential trace elements[1].

Trace elements	Mid-pregnant women			Late pregnant women			Lactating women		
	EAR	RNA	UL	EAR	RNA	UL	EAR	RNA	UL
Cu, mg/d	0.7	0.9	8.0	0.7	0.9	8.0	1.1	1.4	8.0
Fe, mg/d	19	24	42	22	29	42	18	24	42
Zn, mg/d	7.8	9.5	40	7.8	9.5	40	9.9	12	40
Cr, μg/d	34[2]	NE	NE	36[2]	NE	NE	37[2]	NE	NE
I, μg/d	160	230	600	160	230	600	170	240	600
Mn, mg/d	2[2,3]	NE	11[3]	2[2,3]	NE	11[3]	2.6[2,3]	NE	11[3]
Mo, μg/d	92	110	900	92	110	900	88	103	900
B, mg/d	NE	NE	20[3]	NE	NE	20[3]	NE	NE	20[3]

[1] The values were derived from Chinese dietary reference intakes – Part 3: Trance element (National Health Commission of the PRC. 2017. http://www.nhc.gov.cn/wjw/yingyang/201710/ef2d42ee35894a46b7726457d08d7e2d.shtml, accessed on 7 Jan 2021); [2] Values of adequate intake (AI) were used; [3] The values were derived from Dietary Reference Intakes for Vitamin A, Vitamin K, Arsenic, Boron, Chromium, Copper, Iodine, Iron, Manganese, Molybdenum, Nickel, Silicon, Vanadium, and Zinc (Institute of Medicine (US) Panel on Micronutrients. Washington (DC): National Academies Press (US); 2001). Abbreviations: EAR, estimated average requirement; RNA, recommended nutrient intake; UL, tolerable upper intake level; NE, Not established.

Table S4. Quantile regression results from the adjusted models for median concentrations (ng/mL) of essential elements for pregnant and lactating women [1.]

Characteristics	Adjusted β (95% confidence interval)								
	Cu	Fe	Zn	I	Cr	Mn	Co	Mo	B
**Physiological stage**									
Mid-pregnancy	Ref.	Ref.	Ref.	Ref.	–	–	Ref.	Ref.	Ref.
Late-pregnancy	133 (90 to 176)***	–343 (–408 to –277)***	–129 (–152 to –105)***	–12.1 (–14.8 to –9.4)***	–	–	0.34 (0.25 to 0.44)***	–0.19 (–0.32 to –0.07)**	15.7 (11.0 to 20.4)***
Lactation	–653 (–696 to –611)***	–141 (–206 to –76)***	9 (–15 to 32)	–44.2 (–46.9 to –41.5)***	–	–	0.13 (0.03 to 0.23)*	0.50 (0.37 to 0.62)***	5.1 (0.5 to 9.8)**
**Geographic region**									
Coastland	–	Ref.	Ref.	Ref.	–	Ref.	Ref.	Ref.	Ref.
Lakeland	–	73 (7 to 138)**	–75 (–99 to –52)***	–10.1 (–12.8 to –7.3)***	–	–0.47 (–0.59 to –0.35)***	–0.23 (–0.33 to –0.14)***	0.37 (0.24 to 0.50)***	–37.3 (–42.0 to –32.6)***
Inland	–	238 (173 to 303)***	–238 (–2691to –214)***	–19.7 (–22.4 to –16.9)***	–	–0.73 (–0.85 to –0.61)***	–0.51 (–0.61 to –0.42)***	0.79 (0.67 to 0.92)***	–49.0 (–53.6 to –44.3)***
**Age, years**									
≤25	–	–	–	–	–	–	–	–0.07 (–0.21 to 0.07)	–
26 to 30	–	–	–	–	–	–	–	Ref.	–
31 to 35	–	–	–	–	–	–	–	0.22 (0.09 to 0.35)**	–
**Pre-pregnancy BMI**									
Underweight	–	–	–	–	–	–	–	–	–
Normal weight	–	–	–	–	–	–	–	–	–
Overweight/obese	–	–	–	–	–	–	–	–	–
**Parity**									
Primiparous	–	–	–	–	–	–	–	Ref.	–
Multiparous	–	–	–	–	–	–	–	–0.32 (–0.47 to –0.17)***	–
**Ethnicity**									
Han	–	–	–	–	–	–	–	–	–
Others	–	–	–	–	–	–	–	–	–
**Education**									
College or higher	–	–	–	–	–	–	–	–	–
High school	–	–	–	–	–	–	–	–	–
Middle school or less	–	–	–	–	–	–	–	–	–
**Annual family income per capita, Yuan [2]**									

<30000	–	–	–	–	–	–	–	–	–
30000 to <50000	–	–	–	–	–	–	–	–	–
50000 to <100000	–	–	–	–	–	–	–	–	–
≥100000	–	–	–	–	–	–	–	–	–
**Delivery mode** [2]									
Vaginal delivery	–	–	–	4.2 (1.9 to 6.5)***	–	–	0.13 (0.01 to 0.25)*	–	8.0 (2.0 to 14.0)**
Cesarean delivery	–	–	–	Ref.	–	–	Ref.	–	Ref.
**Feeding practice** [2]									
Exclusive breastfeeding	67 (21 to 112)**	–	–	–	–	–	–	–	–
Partial breastfeeding	Ref.	–	–	–	–	–	–	–	–
**Freshwater aquatic product** [3]									
Lowest and middle tertiles	–	–	–	–	–	–	–	–	–
Highest tertile	–	–	–	–	–	–	–	–	–
**Marine aquatic product** [3]									
Lowest and middle tertiles	–	–	–	–	–	–	–	–	–
Highest tertile	–	–	–	–	–	–	–	–	–
**Mutton** [3]									
Lowest and middle tertiles	–	–	–	–	–	–	–	–	–
Highest tertile	–	–	–	–	–	–	–	–	–
**Formula** [3]									
No	–	–	–	–	–	–	–	–	–
Yes	–	–	–	–	–	–	–	–	–

[1] Estimated from multivariable quantile regression models adjusted for physiologic stage, geographic region, maternal age, parity, ethnicity, education status, annual family income per capita, pre-pregnancy BMI, and dietary intake of aquatic products, mutton, and formula; delivery mode and feeding practice were also included in the models for lactating women. "–" denotes the variables excluded from the models by stepwise selection; [2] Estimated for lactating women (n=407); [3] A total of 1208 participants were included in the analyses for dietary intakes, because 3 missed dietary information; * <0.05; ** <0.01; *** <0.001.

Table S5. Quantile regression results from the adjusted models for median concentrations (ng/mL) of toxic elements for pregnant and lactating women [1.]

Characteristics	Adjusted β (95% confidence interval)					
	Ni	Al	Sb	Hg	Cd	Pb
**Physiological stage**						
Mid-pregnancy	Ref.	Ref.	–	Ref.	Ref.	Ref.
Late-pregnancy	–1.4 (–2.6 to –0.1) **	–8.9 (–12.1 to –5.8)***	–	–0.07 (–0.10 to –0.03)***	–0.08 (–0.13 to –0.04)***	0.06 (–0.05 to 0.18)
Lactation	4.5 (3.2 to 5.8)***	–11.5 (–14.6 to –8.4)***	–	–0.06 (–0.09 to –0.02)**	0.18 (0.13 to 0.22)***	0.26 (0.15 to 0.38)***
**Geographic region**						
Coastland	Ref.	Ref.	Ref.	Ref.	Ref.	Ref.
Lakeland	0.8 (–0.6 to 2.1)	–23.8 (–26.9 to –20.7)***	0.50 (0.31 to 0.68)***	0.19 (0.15 to 0.22)***	0.08 (0.04 to 0.13)***	–0.57 (–0.68 to –0.46)***
Inland	–5.8 (–7.3 to –4.4)***	–33.3 (–36.4 to –30.2)***	0.80 (0.61 to 0.98)***	–0.16 (–0.20 to –0.13)***	0.19 (0.15 to 0.24)***	–0.67 (–0.78 to –0.55)***
**Age, years**						
≤25	–	–	–	–	–	–
26 to 30	–	–	–	–	–	–
31 to 35	–	–	–	–	–	–
**Pre-pregnancy BMI**						
Underweight	–	–	–	–	–	–
Normal weight	–	–	–	–	–	–
Overweight/obese	–	–	–	–	–	–
**Parity**						
Primiparous	–	–	–	–	Ref.	–
Multiparous	–	–	–	–	–0.08 (–0.13 to –0.03)**	–
**Ethnicity**						
Han	–	–	Ref.	–	Ref.	–
Others	–	–	0.45 (0.09 to 0.81)*	–	0.12 (0.03 to 0.21)**	–
**Education**						
College or higher	–	–	–	–	–	–

High school	–	–	–	–	–	–
Middle school or less	–	–	–	–	–	–
**Annual family income per capita, Yuan**[2]						
<30000	–	–	–	–	–	–
30000 to <50000	–	–	–	–	–	–
50000 to <100000	–	–	–	–	–	–
≥100000	–	–	–	–	–	–
**Delivery mode**[2]						
Vaginal delivery	–	–	–	–	–	–
Cesarean delivery	–	–	–	–	–	–
**Feeding practice**[2]						
Exclusive breastfeeding	–	–	–	–	–	–
Partial breastfeeding	–	–	–	–	–	–
**Freshwater aquatic product**[3]						
Lowest and middle tertiles	–	–	Ref.	Ref.	–	–
Highest tertile	–	–	–0.38 (–0.56 to –0.20)***	0.06 (0.03 to 0.09)**	–	–
**Marine aquatic product**[3]						
Lowest and middle tertiles	–	–	Ref.	Ref.	–	–
Highest tertile	–	–	0.24 (0.05 to 0.43)*	0.05 (0.02 to 0.08)**	–	–
**Mutton**[3]						
Lowest and middle tertiles	Ref.	–	–	–	–	–
Highest tertile	–2.2 (–3.6 to –0.7) **	–	–	–	–	–
**Formula**[3]						
No	–	–	–	–	–	–
Yes	–	–	–	–	–	–

[1] Estimated from multivariable quantile regression models adjusted for physiologic stage, geographic region, maternal age, parity, ethnicity, education status, annual family income per capita, pre-pregnancy BMI, and dietary intake of aquatic products, mutton, and formula; delivery mode and feeding practice were also included in the models for lactating women. "–" denotes the variables excluded from the models by stepwise selection; [2] Estimated for lactating women (*n*=407); [3] A total of 1208 participants were included in the analyses for dietary intakes, because 3 missed dietary information; * <0.05; ** <0.01; *** <0.001.
